# Isolated Extensor Hallucis Longus Compartment Syndrome: A Case Report

**DOI:** 10.7759/cureus.51772

**Published:** 2024-01-06

**Authors:** Daniel P McKenna, Cathal McCarthy, Tony Higgins

**Affiliations:** 1 Surgery, Royal College of Surgeons in Ireland, Dublin, IRL; 2 Trauma and Orthopaedics, University Hospital Kerry, Tralee, IRL

**Keywords:** hallucis, extensor, isolated, syndrome, compartment

## Abstract

We present the case of an isolated extensor hallucis longus compartment syndrome following a diaphyseal fibular fracture. Our subject underwent syndesmotic fixation and experienced ongoing pain post-procedure. This was associated with an isolated loss of power in extension of the hallux. A diagnosis of an isolated extensor hallucis longus compartment syndrome followed. Our case highlights the vulnerability of this muscle belly to ischemia and reiterates the value of complete clinical examination in the postoperative patient.

## Introduction

Ours is a case of an isolated extensor hallucis longus compartment syndrome in association with a diaphyseal fibular fracture. We discuss the clinical course and subsequent investigations that lead to a diagnosis of compartment syndrome requiring revision fixation.

Acute compartment syndrome (ACS) is a surgical emergency characterised by a critical increase in myofascial pressures resulting in decreased tissue perfusion [1]. A prompt and accurate diagnosis is required given that myonecrosis can occur within three hours of the inciting incident [2]. A missed diagnosis of ACS is associated with male gender, younger age, and tibial shaft fractures as the result of penetrating trauma [3].

ACS is a time-sensitive diagnosis, and our case highlights the nuance associated with isolated cases involving extensor hallucis longus. Compartment syndrome involving this muscle belly alone has been identified in only four other previous case reports.

## Case presentation

**Figure 1:**
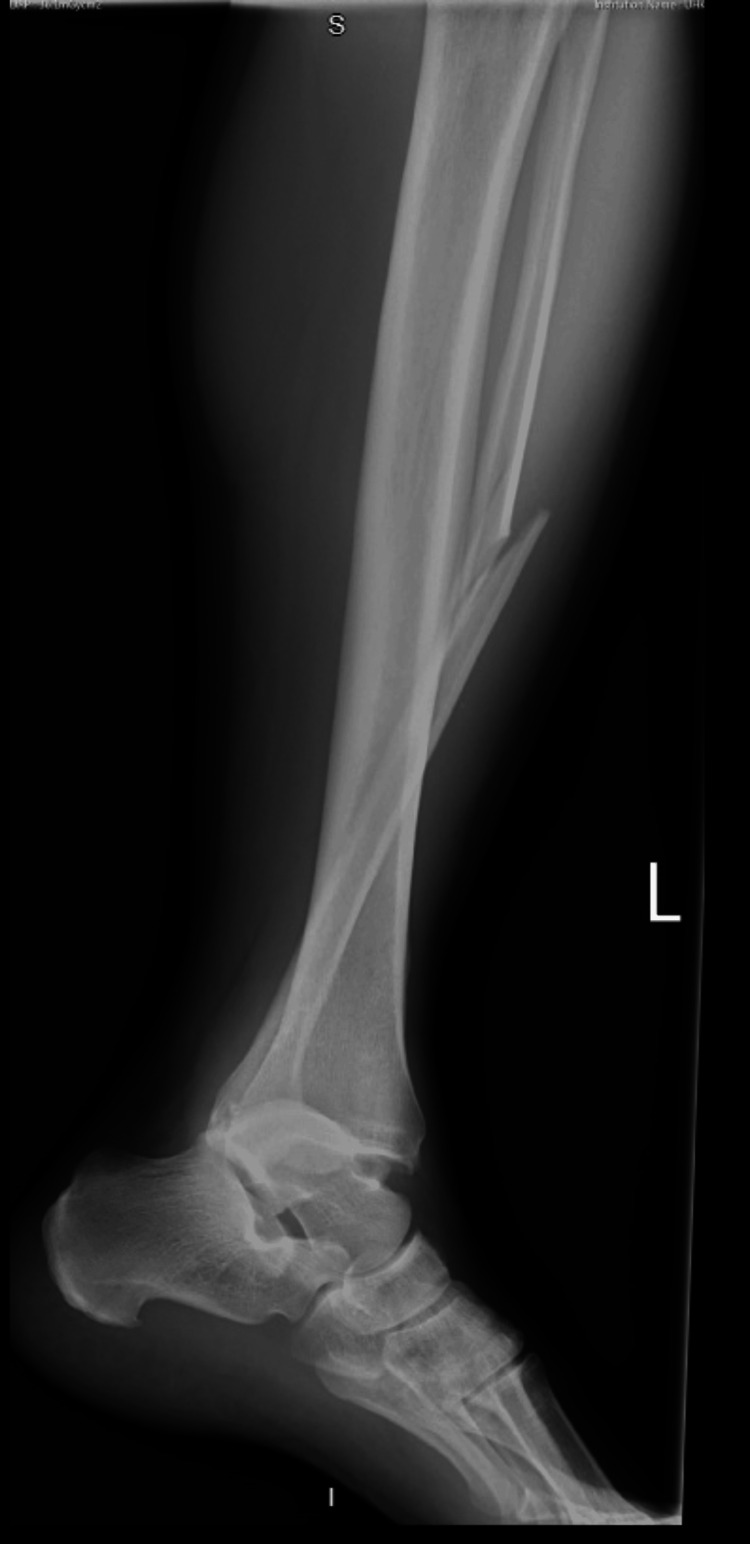
Lateral left ankle radiograph post-tackle playing football

**Figure 2:**
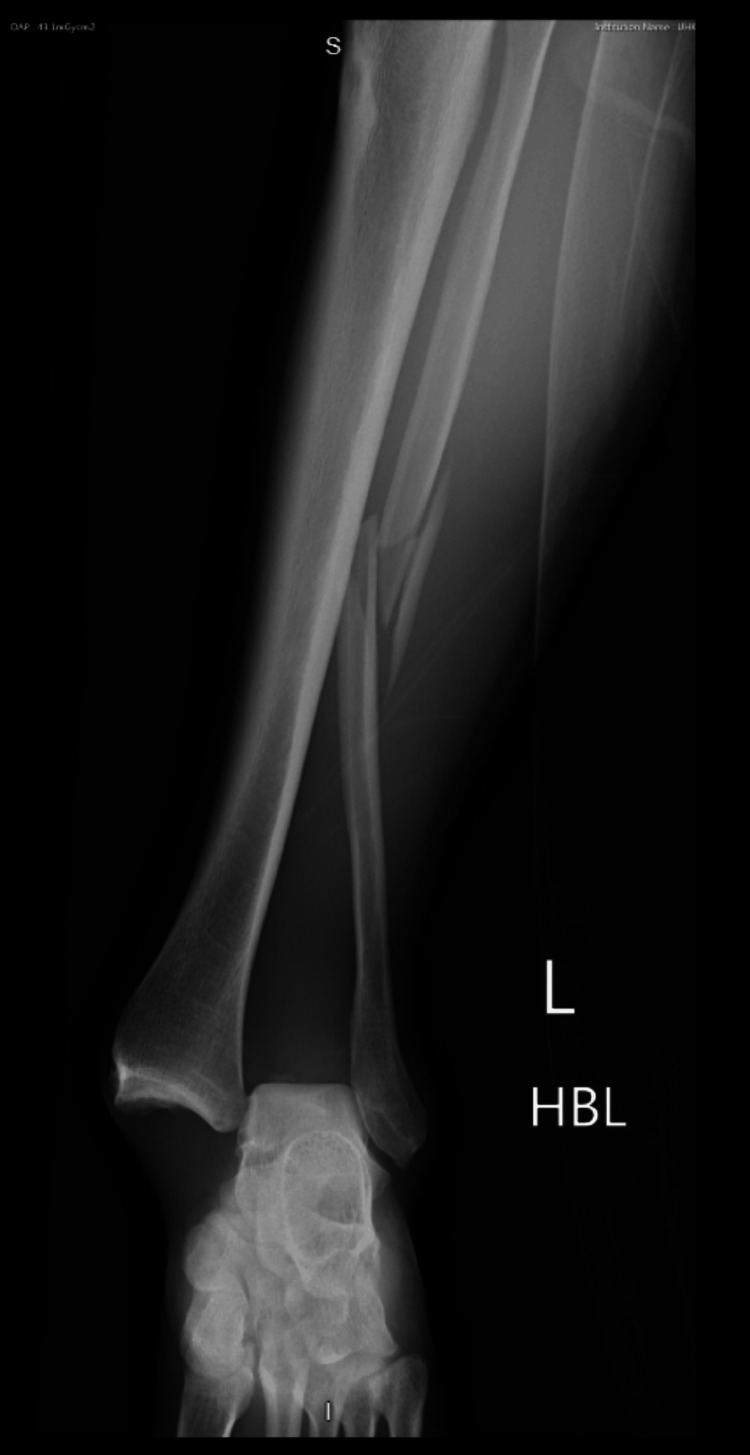
Anteroposterior left ankle radiograph post-tackle playing football

Our subject is a 34-year-old gentleman who sustained a direct blow to the lateral aspect of his left leg during a football match. An obvious deformity was noted, and he presented acutely to the emergency department. Plain films demonstrated a left ankle dislocation with a left fibula diaphyseal fracture (Figures 1, 2).

**Figure 3:**
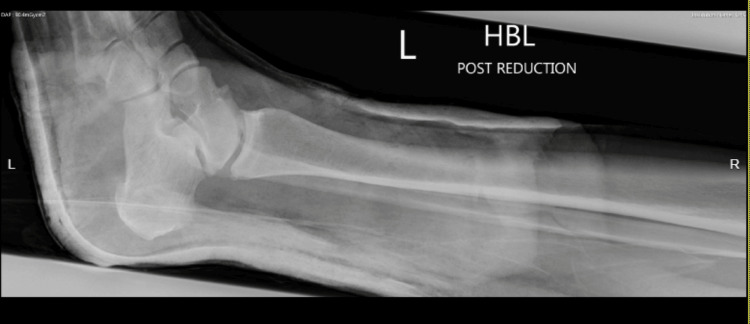
Lateral left ankle radiograph post-reduction under conscious sedation and application of a backslab

**Figure 4:**
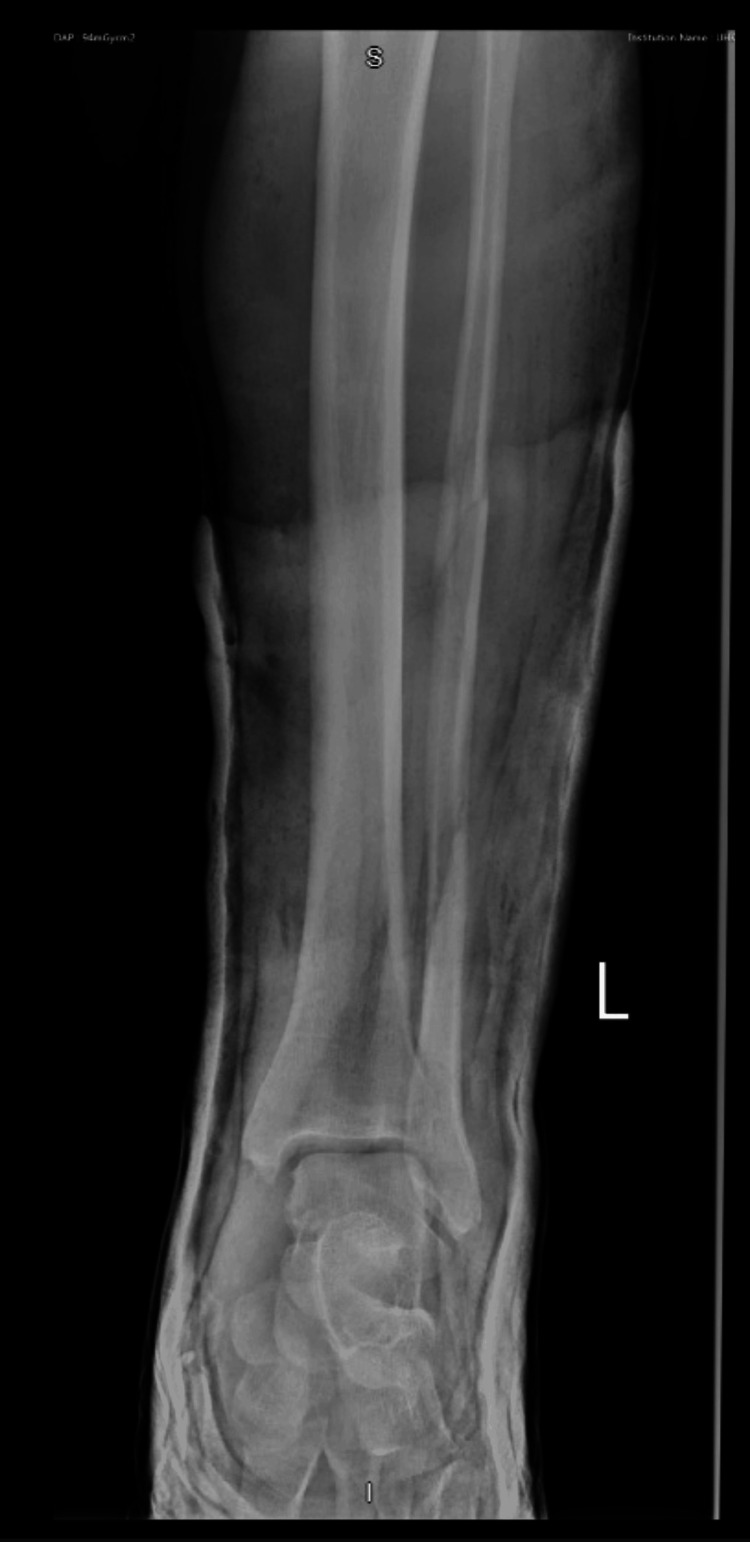
Anteroposterior left ankle radiograph post-reduction under conscious sedation and application of a backslab

This was a closed injury, and reduction was performed in the emergency department under sedation, followed by the application of a below-knee backslab (Figures 3, 4).

There were no neurovascular deficits pre- or post-reduction, and the patient was comfortable post-reduction.

**Figure 5:**
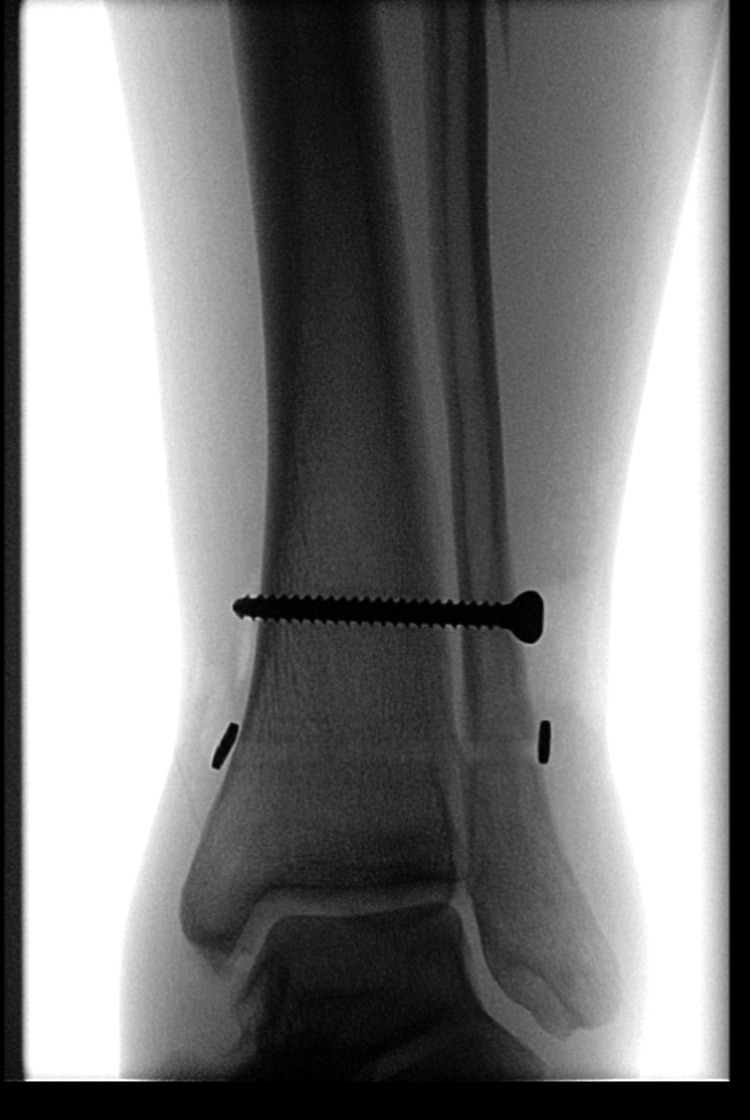
Post-left ankle syndesmosis fixation with a 46 mm cortical screw and ArthrexTM Knotless Syndesmosis TightRope

The following day, under spinal anesthesia, the patient underwent a reduction of the syndesmosis with a pointed reduction clamp and syndesmosis fixation (Figure 5).

Syndesmosis fixation was performed with a 46 mm cortical screw and a suture button fixation using an ArthrexTM Knotless Syndesmosis TightRope (Arthrex, Naples, Florida, United States).

Postoperative analgesia comprised paracetamol, ibuprofen, and opiates in the form of OxyContin 5 mg twice daily. Despite these analgesics, at 36 hours post-fixation, our patient reported severe pain at the anteromedial aspect of his left lower leg. Further opiates were given, and an ultrasound of the leg was unremarkable.

On day three post-fixation, the pain persisted, and a left popliteal nerve block was performed with 20 mL of 0.2% ropivacaine. His creatinine kinase (CK) on this date was 9,764 IU/L. The anteromedial lower leg pain persisted, and the metalwork was removed on day four postoperatively. The reasoning for removing the metalwork was to investigate if this would alleviate our subject's pain symptoms. Intraoperative swabs were taken for culture with flucloxacillin and clindamycin commenced on an empiric basis. Culture swab results were subsequently negative for any growth.

**Figure 6:**
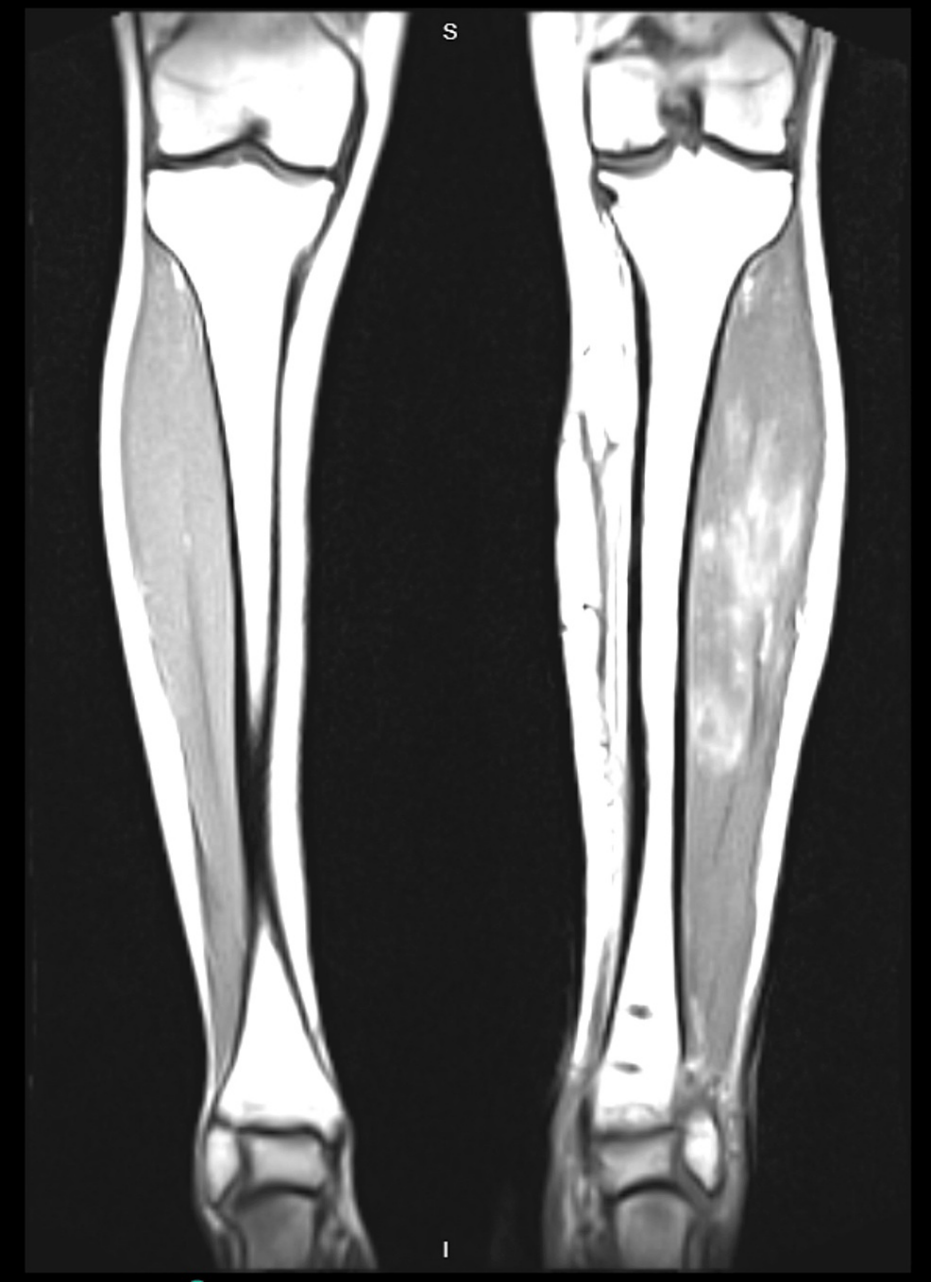
Coronal MRI of left and right legs on day eight post-index procedure. Compartmental oedema of the anterior and deep posterior compartments

**Figure 7:**
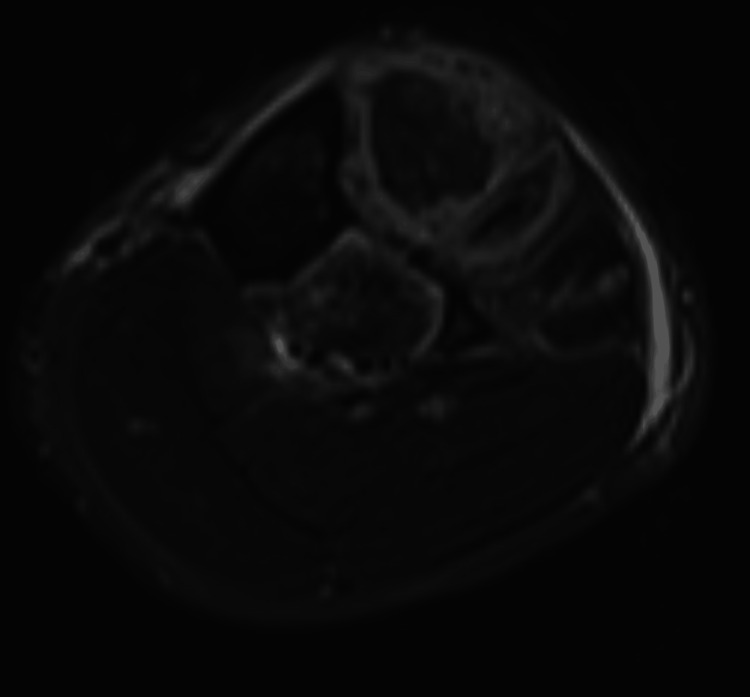
Axial MRI of left leg day eight post-index procedure. Compartmental oedema of the anterior and deep posterior compartments

On day eight, post-index procedure, an MRI of the left lower leg and ankle was performed. This demonstrated compartmental oedema and findings consistent with ACS of the anterior and deep posterior compartments. These findings are shown in Figures 6-7.

**Figure 8:**
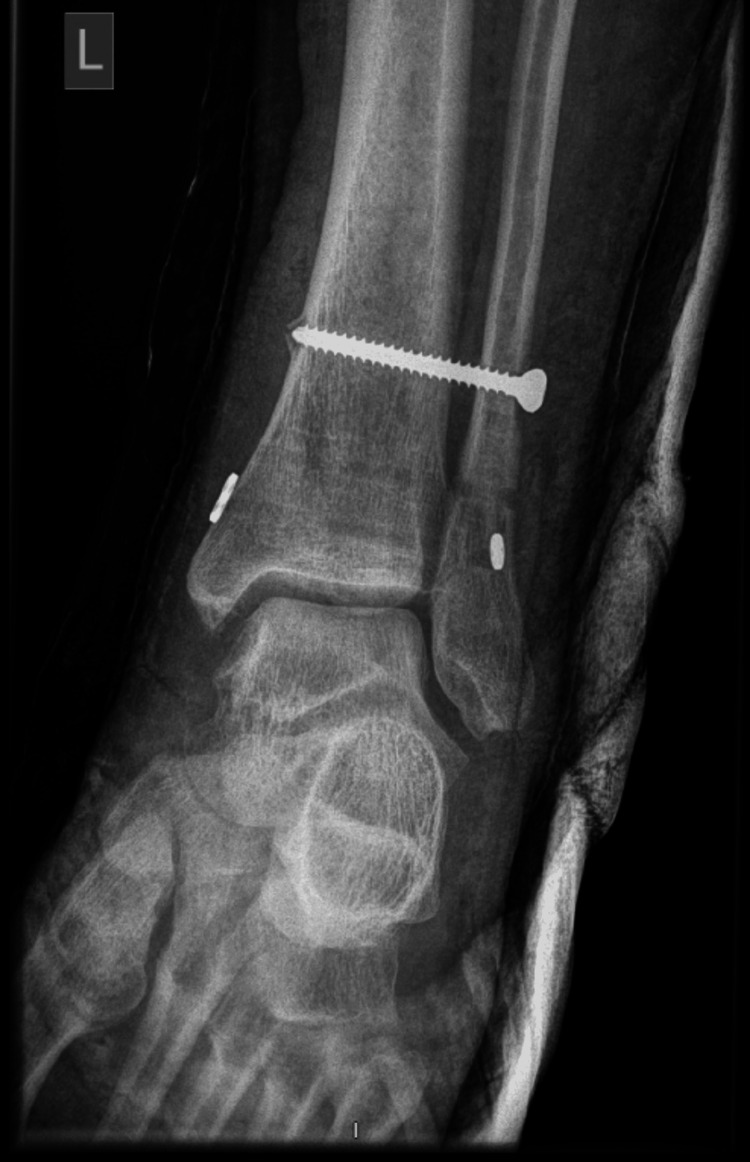
Revision left ankle syndesmosis fixation with 46 mm cortical screw and ArthrexTM Knotless Syndesmosis TightRope

The creatinine kinase on this date had reduced to 1,969 IU/L (local reference range 40-320 IU/L). On examination, our subject had full power in ankle dorsiflexion, isolated weakness of hallux extension, and no sensory loss. Revision syndesmotic fixation with a 46 mm cortical screw, and suture button fixation was performed the following day. The final fixation is shown in Figure 8.

It was decided not to perform fasciotomy during this operation given the patient was now nine days post-index procedure. Concern for wound breakdown given the established tissue necrosis meant that fasciotomy was deemed to be contraindicated. The left ankle pain improved post-fixation, and our patient was comfortable on discharge day two post-revision of his syndesmotic fixation. Our subject was last seen in the outpatient clinic two weeks post-revision fixation. Clinically his only residual weakness remains confined to an extension of his hallux. The plan is to treat any deformity on a symptomatic basis.

## Discussion

The only definitive treatment for ACS is immediate decompression of the compartment in the form of fasciotomy [4]. Our case involves the leg, and, accordingly, a four-quadrant fasciotomy is the standard of care for leg compartment syndrome. Some propose that a single incision can suffice for leg decompression with complication rates equivocal to the standard dual incision technique [5]. However, this evidence is primarily retrospective, and dual incisions remain commonplace. Regardless of technique, fasciotomy is associated with its own morbidities such as pain, muscle weakness, cosmesis, and chronic venous insufficiency [6]. Given that primary closure is contraindicated, infection of the open wounds post-fasciotomy is of concern. Increased body mass index and smoking are recognised risk factors for such infections [7].

In our case, one of the primary areas of interest is his analgesic management. This comprised a spinal block, oral analgesia, and a popliteal nerve block. Nerve blocks are best avoided in closed compartments at risk of raised pressures [8]. However, peripheral nerve blocks have been proven to provide excellent pain relief and decrease hospital stay post-orthopaedic procedures [9]. The ischaemic pain associated with ACS can be distinct from the inciting injury; therefore, patients reporting a sudden increase in pain should raise suspicion. Proponents of regional anaesthesia recommend minimal local anaesthetic usage in cases at risk of compartment syndrome. In theory, this facilitates motor function to remain intact, thereby allowing the detection of breakthrough pain [10]. However, at present, no high-quality evidence exists in favour of or against nerve blocks in orthopaedic patients at risk of ACS [11].

While ACS is primarily a clinical diagnosis, there are numerous biochemical disturbances that can be seen during its progression, and close monitoring of these is imperative for the management of the systemic complications associated with ACS. In our case, we obtained a CK of 9,764 IU/L, whereby a CK in excess of 4,000 IU/L is associated with acute compartment syndrome [12]. Following on from an elevated CK, rhabdomyolysis is of concern for the ACS patient. Patients who are suspected to have ACS should be assessed for rhabdomyolysis and acute kidney [13]. Our patient did not require any renal supportive therapy, likely because of his ACS being primarily isolated to one muscle belly. His creatinine never surpassed 88 µmol/L, and his urea reached a maximum of 4.8 mmol/L during admission. However, this serves as a reminder to consider other systems when treating the ACS patient.

To provide context for our case in the existing literature, a search of MEDLINE was performed. On November 19, 2023, the search criteria of ((compartment syndrome) AND (extensor hallucis longus)) yielded 22 results. A total of four other cases of isolated extensor hallucis longus compartment syndrome were identified by Leung, Wu, and Yoshikawa [14-16]. All of these cases involved a tibial fracture with one having an associated ipsilateral fibula fracture [15]. As in our case, MRI confirmed isolated compartment syndrome of EHL in three of the cases [14,16]. Regarding the anatomy of the EHL tendon, it is hypothesised to be particularly vulnerable to ischaemic insult. Compared to other muscles in the anterior compartment of the leg, its fibres descend deeper under the extensor retinaculum and its vascularity appears more tenuous [17]. This is of note for clinical practice, and a more nuanced clinical exam may be required to detect isolated EHL compartment syndrome.

Post-ACS the four described cases all presented to the outpatient clinic with an extensor contraction deformity to their hallux. All underwent either tendon transfer or z-plasty to the EHL tendon with achieved in each case.

## Conclusions

This case report of clinically isolated extensor hallucis longus compartment syndrome highlights the complexity and potential pitfalls associated with orthopaedic trauma. ACS requires a high index of suspicion for diagnosis, and this can be complicated by analgesic requirements post-injury. Given the vulnerability of EHL to vascular insult, we recommend it be examined distinctly from other muscles in the anterior compartment of the leg.
